# Effects of Alcohol on EEG Activity: A Systematic Review Focused on Sex-Related Differences in Youth

**DOI:** 10.2174/1570159X23666241106095027

**Published:** 2024-11-06

**Authors:** Adrián S. Elliott, Román D. Moreno-Fernández, Patricia Sampedro-Piquero

**Affiliations:** 1 Departamento de Psicología Biológica y de la Salud, Facultad de Psicología, Universidad Autónoma de Madrid, Madrid, Spain;; 2 Facultad de Educación y Psicología, Universidad Francisco de Vitoria, Madrid, Spain

**Keywords:** Alcohol, EEG, EROs, ERPs, youth, sex, systematic review

## Abstract

**Background:**

Most electroencephalographic (EEG) investigations on alcohol have focused on adults, and scarce data is available about the potential of EEG measurements to detect young people at high-risk, as well as, to understand possible sex differences in alcohol impact on the brain.

**Objective:**

This systematic review aimed to explore sex-related differences in EEG among young people with alcohol misuse, alcohol use disorder (AUD), and offspring of families with AUD.

**Methods:**

A systematic review of the literature was conducted following PRISMA guidelines. Review protocol was registered in Prospero (ID: CRD42024511471). After article selection process and quality assessment, 25 studies were included in our review. The search included participants between 12 and 30 years old with problematic alcohol consumption, as defined by DSM, AUDIT, or specific alcohol misuse questionnaires.

**Results:**

It seems that beta was generally higher in young males with AUD, and they usually exhibited greater interhemispheric connectivity (interHC), whereas young females with AUD tended towards enhanced intraHC. P3 appears to be particularly sensitive to alcohol misuse, with males typically exhibiting a lower amplitude than young females. Other event related potentials (ERPs) such as N415, P640, and the error-related negativity (ERN) lacked sufficient methodological support to draw conclusions regarding sex differences, N340 and P540 suggested avenues for expanding research on memory processing, indicating differences in amplitude between males and females.

**Conclusion:**

Considering sex variables in clinical research will enhance our understanding of alterations in brain function and structure with the goal of tailoring treatment strategies for AUD.

## 1 INTRODUCTION

Despite the ongoing debate on nomenclature, people ranging from the age of 10 to 24 are usually referred to in research as ‘young people’, a category that can be subdivided into adolescence and young adulthood, with the 18-20 age range being the limit between the two subsets [1]. Nevertheless, recent studies have suggested that rather than a range of 10-19 years, a definition of 10-24 years corresponds more closely to adolescent growth [2, 3]. This period of life is characterized by the increased likelihood of sensation-seeking and risk-taking behaviour, constituting a vulnerable stage of development, as well as higher rates of injuries, mental health issues, and substance use [4-7]. The involvement in substance use during early or late adolescence has long been a significant health concern due to its predictive association with subsequent drug use in adulthood, as well as other mental and physical health disorders [8-10]. With regard to this, some studies have suggested that the enhanced brain plasticity present during adolescence could lead not only to compensatory responses that help attenuate the severity of drug-related symptoms but also make it more likely for them to engage in drug-taking behavior in the future [11-14]. According to the Global Burden of Diseases, Injuries, and Risk Factors Study (GBD, 2020) [15], which is a standardized and comprehensive assessment of the magnitude of risk factor exposure and attributable burden of disease, alcohol misuse was one of the leading risk factors for those aged between 25-49 years old. Other studies have also found that alcohol misuse is a leading risk factor for disease burden among youth [6, 16]. Furthermore, the World Health Organization (WHO) global status on Alcohol Report [17] has revealed that alcohol is consumed by more than half of the population in the European Region (59.9% of current drinkers), the Americas (54.1%) and the Western Pacific Region (53.8%) and the pattern of drinking behaviour displayed by young people is similar to the general population. In some regions, people aged 20-24 years maybe even more frequent drinkers, which could mean that the highest prevalence of drinking peaks is within this age group. Finally, a more recent report from the WHO [18] has revealed that the per capita alcohol consumption in the European Region, including the European Union‎, is the highest in the world, which has resulted in proportionally higher levels of the burden of disease attributable to alcohol misuse compared with other regions. Therefore, in this scenario, more attention should be paid to this early period of neurodevelopment because the onset of substance use during adolescence and young adulthood has been shown to affect their vulnerable brain, conferring functional consequences throughout life with a negative impact on emotional and behavioural related variables [19-22].

### 1.1 Neurocognitive and Brain Consequences of Alcohol Misuse During Youth

Alcohol is a highly addictive substance that induces depression in the central nervous system (CNS). This drug exerts its effect mainly by neuroreceptors of gamma-aminobutyric acid (GABA), glutamate, and endocannabinoids, causing indirect effects on the limbic and opioid systems, calcium and potassium channels, and on GABA regulated proteins [23]. This widespread effect results in psychomotor and cognitive alterations, such as difficulties in creating new declarative memories and deficits in logical reasoning and motor coordination. Alcohol consumption produces a stimulation of the brain reward system, a fact that may explain the maintenance of its intake and the development of addiction as in other psychoactive drugs [24]. For instance, it has been described that accumbens functional connectivity (FC) with brain areas, such as the supplementary sensorimotor area and right precuneus, mediates sensation-seeking and alcohol misuse in high-risk (HR) youth [25].

Regarding the brain impact of alcohol misuse on the brain, the neurochemical imbalance caused by continued alcohol consumption at the early stages of brain development leads to morphological and functional changes to key structures, such as the hippocampus [26], the prefrontal cortex, and the cerebellum [27]. Hence, several studies have pointed out that drinking alcohol causes substantial structural and functional impairments to the brain of young adult drinkers, with changes generally occurring in the frontal, temporal, parietal, and occipital lobes [28, 29]. This negative effect affects not only the way in which the different brain areas operate but also how brain networks connect with each other in an efficient manner [30]. In this sense, the integrity of brain white matter is also affected, leading to aberrant connectivity between different brain regions, such as the frontal-parietal network [31] or the left prefrontal-parietal-occipital midline circuits [32], among others. This impaired FC has been related to poorer performance in many neurocognitive domains in young people with alcohol misuse [8, 33, 34]. These cognitive deficits are characterized by their broad effect on different areas, such as attention and processing speed, declarative memory, visuospatial skills, and executive functioning [35, 36]. The severity of this impairment is related to an early age of onset or heavy alcohol consumption [37, 38], which makes some of the impairments observed in attention or learning and verbal memory like those found in adults with a diagnosis of addiction [39, 40]. Finally, the alcohol-related brain alterations described have been associated not only with cognitive dysfunction but also with behavioural, emotional, and motivational domains in young drinkers. Concretely, alcohol is also related to increased impulsivity [41], risky behaviour [42], and difficulties in emotional regulation [35, 43].

### 1.2 Sex-related Differences in Brain Structure and Function

Over the past decade, there has been an 84% increase in the rate of AUD among females, compared with a 35% increase among males. Furthermore, the rates of alcohol misuse and HR drinking have risen by 16% and 58% among females, respectively, whereas males have experienced increases of 7% and 16% in these categories [17]. Consistent with these changing patterns of alcohol consumption, recent studies suggest that females are more susceptible than males to alcohol-induced liver inflammation, cardiovascular disease, memory blackouts, hangovers, and certain cancers [44-46]. The role of sex differences in the development of CNS complications associated with alcohol misuse is less clear and needs special attention.

The effect of alcohol on brain structure and function in emerging adults could be different between young males and females, according to results from neuroimaging research and preclinical data using animal models [47]. Regarding the changes in brain structure, Pérez-García *et al*. (2022) [48] found a significant sex interaction in a longitudinal study in whole-brain analysis in humans, revealing smaller right rostral middle frontal gyrus specifically in males displaying binge drinking (BD), but not in the controls or in the females, whereas BDs females showed reduced nucleus accumbens when compared to the other groups. In fact, the results from different sources imply that selective brain areas can be altered in a different way not only depending on sex, but this may also vary in the stage of alcohol addiction: BD/intoxication (basal ganglia and mesocorticolimbic pathways), preoccupation/anticipation (frontal cortex and hippocampus) and negative/affect withdrawal (extended amygdala) [47]. Another whole-brain study, in this case in mice, suggested that the females were associated with a higher risk of alcohol-related brain volume reduction, as measured by longitudinal structural magnetic resonance imaging [49].

With respect to the functional analysis of the brain, a recent research study carried out on 1,359 people has found brain networks that predict vulnerability for future alcohol misuse in young females during both the reward and inhibitory tasks performed using functional magnetic resonance imaging (fMRI), whereas the brain network that succeeded in identifying risk for males was only inhibitory control but not reward [30]. Interestingly, another research carried out using fMRI found that alcohol-naïve young females exhibited higher activation in the frontal areas in response to alcohol picture cues when compared to the males, but the pattern was reversed in the moderate-to-heavy drinking group, with males displaying greater activation than the young females in response to alcohol picture cues [50]. In recent years, several studies have made interesting approaches to the study of the effects of alcohol abuse by considering variables such as the sex or age of the participants and by using different methodologies related to brain activity such as positron emission tomography (PET) [51], magnetic resonance spectroscopy [52], or diffusion tensor imaging [53].

All in all, the alterations that alcohol misuse induces in the brain structure and function should lead to the search for brain biomarkers of at-risk youth, as well as the development of early interventions to prevent progression to addiction or further cognitive impairment. While structural imaging techniques like MRI and functional methods like PET offer valuable insights into brain structure and activity, EEG is a non-invasive and cost-effective technique that provides a unique window into the dynamic electrical activity of the brain in real-time. Hence, we can obtain accurate information about brain activity during cognitive performance tasks or drug-related stimuli. There are also previous reviews that have been focused on different functional neuroimage findings [54] or brain structural alterations [55]. Hence, we consider that an updated review exploring only the EEG technique can be very useful for researchers and experts specialized in this field, particularly when sex-related differences are not usually addressed.

In the following section, we will highlight the potential of EEG methodology as a non-invasive tool that enables us to elucidate the alterations in brain electrical activity, which can help us to detect risk patterns even considering the particularities that have been shown to influence the alcohol consumption and progression, such as the sex of the participants.

### 1.3 EEG Biomarkers and their Clinical Implications

Electrophysiological techniques have demonstrated their efficacy in the understanding of neurocognitive function and the underlying dysfunctional processes associated with diverse psychiatric conditions, such as AUD [56]. EEG offers several methodological and analysis approaches because resting EEG is a promising biomarker of the neurotoxic effects of substance use. The effects of alcohol consumption in young people on resting brain activity are still poorly understood, but the limited studies that have been carried out seem to indicate that they share the same alterations as those described in adults (such as increased theta (4-8 Hz) and/or beta (13-21 Hz) power) [57-59]. Together with these findings, the relative increase in fast-beta power has shown to be a biomarker for potential future AUD, even in the absence of familial AUD in young BDs [60]. On the other hand, a growing number of studies have suggested that the theta/beta ratio (TBR) is inversely related to executive cognitive control, representing a measurement of cognitive processing capacity [61, 62], and it has also been associated with anxiety traits and resilience [63, 64]. Consequently, it would appear that the assessment of this biomarker could shed light on the detection of cognitive deficits in both clinical and at-risk populations [35]. Although the functional significance of heightened beta and theta rhythms in individuals with alcohol misuse remains the subject of debate, the elevated power within these frequency bands is frequently interpreted as indicative of cortical hyperexcitability (for beta rhythms) and diminished cognitive processing capacity (for theta frequencies) [65, 66]. Other EEG measurements, such as FC or spectral power and network analyses, have shown that substance abuse alterations usually express themselves through neural hyperactivation and decreased interregional neural communication [56].

On the other hand, problematic alcohol consumption and related variables have also been shown to affect neuroelectric measures related to cognitive processing (ERPs). The P3 potential is an ERP usually studied through oddball paradigms, and it is understood to be a neural correlate of decision-making and signal matching [67, 68]. Considering this, this evoked potential has become a common ERP in the field of alcohol research, and deviations in parietal P3 and mid-frontal theta have shown to be present prior to the initiation of alcohol misuse and prospectively predicted non-pathological drinking in a large epidemiologically representative adolescent sample [69]. Another longitudinal study revealed that the amplitude of NoGo-P3 in the frontal region (Fpz electrode) was found to be larger in young people who maintained BD for two years but intermediate in the group of subjects that stopped that pattern of alcohol misuse for those two years (ex-BDs), as compared to the control group [70]. This suggests that once the alcohol misuse is reduced, the requirement for heightened neural recruitment or ‘neural effort’ for the inhibitory control is readapted and reduced, but still different from the non-consuming group [70]. Thereby, EEG measures could provide one of the most powerful forecasts for future alcohol consumption in young individuals [71].

### 1.4 Aim and Hypothesis

The main objective of this systematic review is to explore EEG-related differences in young people with alcohol misuse, AUD, or HR consumption, considering the relevance of sex as a mediating variable in its effects. It was hypothesized that alcohol could impact differently in males and females, and EEG methodology could help us to detect young people at risk or assist in the design of more personalized treatments. In addition, the increase in drinking among females underscores the critical need to identify underlying brain mechanisms that may contribute to problematic drinking in order to ultimately develop sex-appropriate treatments for alcohol use disorder [72]. Females may develop more alterations because they achieve higher blood alcohol concentration (BAC) for any given dose of alcohol [73]. Furthermore, methodological problems could also underly the difficulty in detecting sex-related differences. Almeida-Antunes *et al*. (2021) [74] have pointed out that most studies do not report differences between the sexes because drinking characteristics are not displayed separately for males and females. Apart from differences in the physiological effects of alcohol in both sexes, we must also consider the distinct motives that lead males and females to drink alcohol. Thus, recent research suggests that while males usually drink to enhance positive mood states, females often drink to avoid negative mood states [75, 76]. Taking these variables and limitations into account, the various EEG measurements that could be sex-specific in young alcohol consumers, such as resting-state signals or event-related potentials, are explored and discussed in this review.

## 2 MATERIALS AND METHODS

The methods used in this systematic review follow the Prepared Items for Systematic Reviews and Meta-Analysis (PRISMA) guidelines [77, 78]. This review protocol is registered in Prospero (Registration Number CRD42024511471).

### 2.1 Data Sources and Search Strategy

We only considered publications in English and peer-reviewed journals indexed in Journal Citation Reports (JCR), and the search was time limited between 2004 and 2024 in Web of Science, PubMed, Scopus, and PsycINFO under the following key frames and key words: (EEG OR electroencephalog* OR ERP OR event-related oscillation (ERO) OR resting state) AND (young OR youth* OR adolescent* OR teen*) AND (alcohol* OR ethanol) AND (sex). The search included human empirical studies, participants between 12 and 30 years old with problematic alcohol consumption as defined by DSM, AUDIT, or specific alcohol misuse questionnaires. In addition, the studies must have had a control group without an AUD diagnosis or any substance use disorder. Consumption of other drugs was an exclusion criterion, but not comorbidity in the sample (*i.e*., anxiety or depression). Finally, all the studies included were aimed at determining the neurofunctional impairments associated with problematic alcohol consumption assessed by EEG or ERP. Following these inclusion and exclusion criteria, a total of 434 articles were found.

### 2.2 Article Selection Process and Quality Assessment

All the authors of this paper used the Covidence systematic review software (Veritas Health Innovation) for the selection process. After removing 163 duplicate articles, a total of 271 were screened, of which 224 were deemed irrelevant. The remaining 47 articles were independently assessed by the authors of this systematic review, and following discussion and consensus, it was found that 25 of those studies were eligible for the present review. This procedure and a summary of the exclusion criteria can be seen in Fig. (**1**). Some of the exclusion criteria include studies that used fMRI, MEG or researched with older demographics. With the aim of assessing the quality of the selected publications, an examination according to the standards of the NHLBI Quality Assessment Tool for Observational Cohort and Cross-Sectional Studies was implemented independently by two of the authors, A.S.E. and R.D.M.-F., and later discussed and agreed upon. (Heart and Lung and Blood Institute (NHLBI, 2021) (Table **1**).

## 3 RESULTS

### 3.1 Main Findings

The database search resulted in the identification of 434 articles. From these, 163 duplicated papers were excluded using Covidence software (Covidence systematic review software, Veritas Health Innovation, Melbourne, Australia). The titles and abstracts of the remaining 271 articles were scrutinized, and 224 studies were excluded. In the case of any doubt, the manuscripts were submitted for full-text reading. Following the final screening of 47 full texts, a total of 21 studies were excluded for not fulfilling our criteria. Finally, 25 manuscripts were included, with a total of 12561 individuals, of which 52.66% were female.

### 3.2 Study Characteristics and Quality Assessment of the Studies

Most of the studies included in this review (68%) were published after 2010, and 12% were published between 2021 and 2023. More than half of the studies (72%) were conducted in the United States, 8% in Belgium, 12% in Australia, and the remaining 4% in Spain and 4% in the United Kingdom. Three studies (12%) were longitudinal. The studies’ samples were composed mostly of young people over 18 years old, *i.e*., late adolescence. EEG rhythms during resting-state were assessed in some of the studies (28%), while the remaining manuscripts evaluated different cognitive processes, such as inhibitory control by a Go/NoGo task, attention by Visual Signal Task, or the oddball paradigm, or emotional functions through facial discrimination, among others (72%). Even more variability was found in the remainder of the studies in which different paradigms were used including Iowa Gambling, Flanker, and the Rey Auditory Verbal Learning Test (RAVLT). From the remaining studies reviewed, different ERPs were explored, together with several ERO analyses, all of whose results are thoroughly detailed in the following sections. Finally, regarding quality assessment by the NHLBI criteria, twenty of the studies included were deemed to be of a high (44%) or intermediate quality (36%), while the five studies were rated as being of poor quality (20%). The main limitations of the studies were the lack of justification of the sample size, not considering the different levels of the variables of interest, the insufficient time employed to see any effect or the lack of statistical control over confounders.

### 3.3 Sex-related Differences in EEG Resting-state

**Fig. (2):**
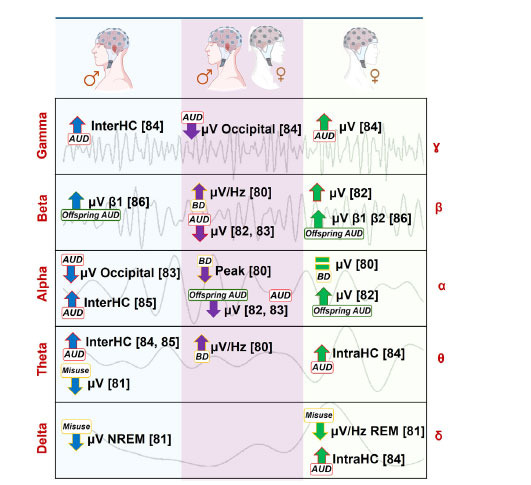
Summary of EEG resting state findings considering sex-related differences and the relationship with alcohol (offspring, misuse, BD, or AUD) in young people in each frequency band. In the left and right columns, in blue and green, the differential EEG measure is represented in the alcohol use groups of males when compared to the females, or *vice versa*, respectively. The column in the middle shows the studies selected that observed differences in the non-consuming control group.

Out of the six articles employing a resting state approach, only one, the study by Affan *et al*. (2018), reported no sex-related differences [79]. Additionally, a seventh article using polysomnography was included in this section, and although it differed from the rest of the EEG resting state studies (Fig. **2**), we will also mention its main results here [80].

In general, the revised evidence found that alcohol misuse was associated with a significant change in spectral power and connectivity (Table **2**). Specifically, a decrease in alpha spectral power was commonly observed, occasionally extending to beta frequencies [81, 82]. However, research carried out by Ehler *et al*. (2004) [81] found that low alpha and beta power varied as a function of sex. Females were found to have a significantly higher EEG power in the slow alpha frequencies in both beta frequency bands (12-20 Hz, 20-50 Hz). Parallel to this, it was also described that AUD was significantly associated with lower alpha levels in the right and left occipital areas in males, but this result was not found in females [82]. A study by Kinreich *et al*. (2021) [83] determined that alcohol consumption was also linked to reduced occipital gamma activity, with notable sex-related differences, such as a higher interHC gamma coherence in males and greater gamma amplitude in females. Furthermore, the males exhibited increased theta interHC, while the females showed a higher intraHC theta and delta activity. Other results, such as those of Meyers *et al*. (2019) [84], observed an elevated interHC alpha and theta connectivity in males. On the other hand, Rangaswamy *et al*. (2004) [85] suggested that HR males were characterized by a higher beta1 activity (12-16 Hz), whereas HR females presented higher beta2 (16-20 Hz) and beta3 (20-28 Hz) activity as compared to the low-risk (LR) participants. Moreover, increased beta2 and beta3 power was also observed in HR females only when they had two or more AUD first-degree relatives.

Finally, the polysomnographic research by Kiss *et al*. (2023) [80] found alcohol effects related to a lower delta power in the NREM sleep phase, especially in the male participants, as well as a lower theta power when a moderate/heavy drinking pattern was present in the males. However, the females presented a rapid decline in delta power during REM sleep compared with the young males.

### 3.4 Sex-related Differences in ERPs and EROs Associated with Cognitive Processes

A total of eighteen articles used ERPs or EROs within experimental paradigms, such as the oddball task, the Eriksen flanker task, or the gambling task. The ERPs commonly analyzed included the P3, together with P1, N2, P450, late 400ms wave (N4S), N415, P640, N340, P540, and ERN. The most explored ERO was theta, together with alpha and beta. Overall, these event-related studies frequently uncovered significant effects of HR-alcohol misuse or AUD, but sex-related effects varied considerably among the studies.

Among the 18 studies, 10 analyzed the P3 component. Blanco-Ramos *et al*. (2019) [86] discovered significant differences only in the female's responses to alcohol-related stimuli, with no notable effects observed in the males. Kamarajan *et al*. (2015a) [87] reported higher P3 amplitudes in males during a gambling task, except in the frontal lobe, where the females exhibited a higher P3. Magee *et al*. (2023) [88] observed an attenuated P3 among depressed non-AUD, similar to non-depressed drinkers, with insignificant sex differences. Similarly, Maurage *et al*. (2009) [89] did not find any sex differences in emotional P3 latency related to alcohol effects. However, Petit *et al*. (2013) [90] noted an increased P3 reactivity towards alcohol *versus* non-alcohol images only in males. Smith *et al*. (2015) [91] did not find any sex differences in P3 as an index of inhibition, but the females exhibited longer latencies for failed inhibitions (Smith *et al*., 2016) [92]. Perlman *et al*. (2009; 2013) [93, 94] investigated the P3 component, and although a reduction in P3 amplitude was related to problematic substance use, no sex-related effects were observed. Finally, Yoon *et al*. (2006) [95] found that P3 amplitude in both males and females was strongly influenced by genetic factors, especially for the males, whereas the females were mostly influenced by the shared environment, a factor that does not present in the males, who generally had smaller P3 amplitudes.

Other ERPs studied included P1 (2 studies), N2 (3 studies), P450 (1 study), late 400 ms wave (N4S) (1 study), and ERN (2 studies). No sex differences were found for P1 during a Go/NoGo task with alcohol and non-alcohol-related stimuli [86] or during an emotional auditory task [89]. The N2 potential also did not show any significant differences between the sexes in a Go/NoGo task [86], although an emotional variant of this task revealed interactions for certain drinking behaviour, but not for sex differences [88]. Smith *et al.*, (2015) [91] found significant sex-related differences in N2 during an Eriksen flanker task, with larger N2 amplitudes for conflict adaptation in the female heavy drinkers compared to the males. The P450 potential, studied through a facial discrimination task, showed significantly diminished responses in males with AUD when compared to females [96]. Ehlers *et al*. (2011) [97] observed higher amplitude N4S responses in females to acoustic startle stimuli. Smith *et al*. (2017) [98] examined N415, P640, N340, and P540 potentials, with only the latter two compared between males and females. For the N340 potential, the males, regardless of alcohol intake, exhibited larger amplitudes to previously presented words (old words) compared to the females, who showed larger amplitudes to newly presented words. Larger P540 amplitudes were found in the females compared to the males. Finally, ERN was also investigated through a visual stop-signal task, where an alcohol effect was found, irrespective of the participants' sex [98].

Five different studies were reviewed regarding the EROs. Barret *et al*. (2004) [99] found that, unlike their earlier findings in males, there were no significant differences between the conditions for 4- to 11-Hz power, but females generally showed much more beta (14-26 Hz) power during the two experimental alcohol conditions. Ehlers *et al*. (2019) [100] observed that despite the lack of significant differences in ERO energy in response to alcohol-related stimuli in any frequency range when comparing AUD *versus* controls, females had significantly higher energy values than males in the delta, theta, and beta frequencies. The results obtained by Harper *et al*. (2018) [101] found that females exhibited greater ERO power than males, a result qualified by a significant interaction between the drinking index scores and sex, suggesting that the negative association between alcohol misuse and no-go theta power was greater for females. Huang *et al*. (2018) [102] assessed affective processing through a task in which young BDs provided subjective ratings of emotionally evocative images with negative, positive, erotic, and neutral themes. Although BD was associated with lower theta responsivity to emotions, sex-related differences were not found in event-related theta. Finally, Kamarajan *et al*. (2015b) [103] examined ERO theta power during reward processing as well as impulsivity in adolescent and young adult subjects at HR for AUD. The younger males had a significant increase in theta power when compared to the younger females, while the older females showed higher theta power than the older males. A significant sex difference was observed only in the loss condition during the monetary gambling task, in which the males showed higher theta power than the females [109, 110].

## 4 DISCUSSION

This systematic review aimed to explore sex-related differences in EEG in young people with alcohol misuse or AUD. Furthermore, we will display possible patterns of sex differences in resting state or in ERPs and EROs responses according to the risk of developing alcohol misuse in offspring of families with AUD, the level or severity of alcohol misuse (*i.e*., binge drinking or heavy consumption), or the presence of AUD diagnostic criteria.

### 4.1 Resting state EEG Findings

The resting state results showed several similarities among the studies but also important differences, probably due to the high variability in the young sample analysed in the different studies (*e.g*., BD [79], AUD [82, 83, 84], family history, but no personal history of AUD [81, 85]). In general, all the studies found alcohol-related changes in EEG, but sex-related effects were not observed in all the research [79].

#### 4.1.1 Offspring of Families with AUD

Ehlers *et al*. (2004) [81] and Rangaswamy *et al*. (2004) [85] analysed sex-related differences in a sample of young males and females with families with AUD but who did not present a personal history of alcohol misuse. Both observed that the young females exhibited higher activity in certain brain frequency bands, specifically in the alpha and beta bands, compared to the males. Together with this shared finding, the HR females also showed increased activity in the beta 2 and beta 3 bands if they had two or more AUD first-degree relatives [85].

#### 4.1.2 Alcohol Misuse: BD or Heavy Consumption

When alcohol misuse is present in the sample in a BD way, changes in fast beta activity have also been described in young BDs, as well as in individuals with AUD, so it could constitute a biomarker for potential future AUD, even in the absence of familial alcohol problems [61]. The beta band is generally understood to be an index of alertness and increased arousal, so, from a pathophysiological perspective, a higher activity of beta could also result in an abnormally strong inhibition of behavioural and cognitive changes especially in females [104].

#### 4.1.3 AUD

Regarding the neurobiological origins of beta power in relation to AUD, increases in this activity have generally been associated with enhanced cortical excitability due to GABAergic dysfunction, which underlies their predisposition to AUD [105-107]. Kinreich *et al.* (2021) [83] and Meyers *et al*. (2019) [84] revealed that young males with AUD had higher interHC theta connectivity. In the study carried out by Kinreich *et al*. [83], this increased interHC theta connectivity was found in the occipital, frontal, and temporal lobes, while the females showed higher slow wave intraHC (delta, alpha) in the frontal-parietal and temporal-parietal lobes. Increased frontocentral, tempo-parietal, centro-parietal, and parietal-occipital interHC theta and alpha connectivity was also revealed by Meyers *et al*. (2019) in males with AUD from the ages of 18-31 [84]. With regard to these results, several studies have indicated that increased theta connectivity does not indicate a better FC, but rather it has been proposed as a compensatory mechanism for impaired areas or reduced or altered connectivity [31, 108].

We also found studies with contradictory results, which could be explained in part due to methodological differences. Thus, according to Kinreich *et al.* (2021) [83], females with AUD had a higher delta power, but Kiss *et al*. (2023) found a lower delta power [80]. This difference could be understood by considering that the experimental approach performed by Kiss *et al*., which consisted of a polysomnographic study and the significant results were observed in the frontal and the occipital lobes, while Kinreich *et al*. [83] pointed to the frontal and parietal areas. Furthermore, the sample analysed in both studies was not comparable. Kinreich focused on young people with AUD, while in the study by Kiss *et al.* (2023) [80], although the young people presented different levels of alcohol consumption, they did not meet the diagnostic criteria for an AUD. Finally, in the studies of Kinreich *et al.* (2021) [83] and Meyers *et al*. (2019) [84], contradictory results with respect to alpha frequencies were also revealed. Thus, while in one of the studies, the females with AUD showed higher slow alpha wave intraHC in the -parietal and temporal-parietal lobes [83], the other research revealed higher alpha connectivity in the young males when compared to the females [84]. Ehlers and Philips (2007) [82] observed that it was AUD and not a family history of addiction, which was associated with lower spectral power in the alpha frequency range in the right and left occipital areas in the young males but not in the females.

Therefore, despite the discrepancies among the studies, which can be attributed to methodological differences and sample characteristics, it seems that based on the results reported, beta was generally higher in the young males with AUD, and they usually exhibited greater interHC, in contrast to the AUD females who tended toward enhanced intraHC. Nevertheless, future studies are required to further validate these results with larger cohorts, as well as ensure sampling uniformity.

### 4.2 ERPs and EROs Results

#### 4.2.1 Offspring of Families with AUD

Sex differences in the context of alcohol misuse or in HR offspring from families with AUD were found regarding P3 potential. Although both the HR males and females manifested lower P3 amplitudes, this deficit became augmented in the males as they aged during development, but it got attenuated in the females as they aged into late adolescence and young adulthood [87]. This sex-specific result was in line with previous results indicating that low P3 amplitudes in HR offspring of individuals with AUD have been reported more often in males than in females [110]. Interestingly, this lower P3 amplitude was negatively correlated to impulsivity score, especially in males [111, 112].

**Fig. (3):**
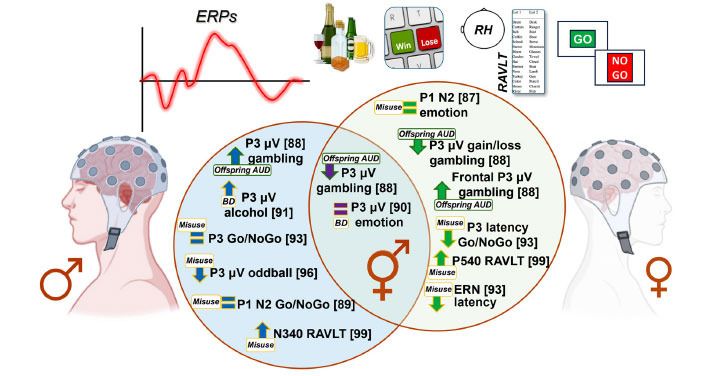
Visual representation of ERP findings considering sex-related differences in young people and the relationship with alcohol (offspring, misuse, BD, or AUD).

Regarding the ERO results, the HR offspring of families with AUD showed significantly lower theta power than the LR subjects, and the differences were more robust among males during a monetary gambling task [103]. This finding suggested that ERO theta power during reward processing in HR individuals, along with personality characteristics, such as impulsivity, could act as a potential factor for a vulnerability to develop AUD. Moreover, in this reward processing protocol, it was also found sex-related differences in reward processing. Thus, only in the loss condition did young males show higher theta power than young females in frontal and central regions [103]. On the other hand, females, especially the HR females during the gain condition, exhibited significantly increased theta power compared to their male counterparts (Fig. **3**). These results seem to suggest differences across development related to the sex in ERO responses, which has also been observed in different frequency bands to cognitive and emotional processing [112-115]. Therefore, additional research investigating the developmental paths of reward-associated ERO theta power during youth in males and females could provide further insights into elucidating these results.

#### 4.2.2 Alcohol Misuse: BD or Heavy Consumption

When alcohol misuse is present in a BD way, BD males had a higher P3 amplitude for alcohol stimulus, probably related to a greater attentional bias towards them, but no sex differences were found regarding P3 latency [90]. On the other hand, when this alcohol misuse is heavy regularly, females manifested shorter P3 latencies for the Go/NoGo task when compared to males, but no sex differences were found for P3 amplitude in a Go/NoGo task [92]. This result could be related to the worst performance of the females in the inhibition task because P3 is commonly understood as an endogenous potential involved in decision-making and signal matching [116]. Moreover, reduced P3 amplitude is considered an indicator of the broad neurobiological vulnerability that underlies disorders within the externalizing spectrum, such as future AUD, substance use disorder (SUD), conduct disorder, or antisocial behaviour, among others [117]. Finally, it has also been shown that the P3 amplitude is strongly associated with genetic factors, especially in males [95]. Additionally, this study also found that young males who presented with alcohol misuse had a smaller P3 amplitude in the rotated-heads oddball paradigm, and this effect was more prominent than in females [95].

Concerning the P1 and N2 potential, no differences based on sex were found in an alcohol-related Go/NoGo task [86, 88] nor in an emotional valence judgment task [89] in a sample of BDs. The P1 potential is a perceptual index sensitive to the physical characteristics of the stimuli and attentional influences [118], whereas the N2 potential is usually associated with the conflict monitoring process by Go/NoGo tasks [119, 120]. The lack of differences in these earlier stages of stimulus processing could be due to the cognitive task applied. Thus, alterations in these ERPs have mainly been described in cue reactivity tasks that usually consist of the passive visualization of substance-related stimuli [68]. Finally, N340 and P540 were only studied by Smith *et al.*, (2017) [97], who revealed sex-related differences for both measurements. In this study, the RAVLT was applied, and the results showed that males who were heavy drinkers displayed larger N340 amplitudes for previously presented words (List A), while the females presented larger amplitudes for newly presented words (List B). Concerning P540, the females had larger amplitudes than the males. This could mean that the males who were heavy drinkers but did not fulfill AUD criteria may present difficulties with familiarity-based recognition, as seen for the N340 amplitude, and the heavy-drinking females could need greater use of recollection-based recognition, as seen in the larger amplitude of P540.

The study by Harper *et al.* (2018) aimed to assess the potential causal effect of normative levels of drinking on EEG correlates of response inhibition in a population-based sample of 24-year-old same-sex twins [101]. Their results suggested that heavier alcohol consumption was associated with diminished mid frontal theta power and medial frontal cortex - dorsal prefrontal cortex theta-band FC during the demands of response inhibition. This association was moderated by sex, such that the relationship between drinking and theta-band dynamics was greater for females, which could indicate that females are at a higher risk of developing alcohol-related brain dysfunction [101]. Regarding this hypothesis, other studies have observed that brain and cognitive alterations progress faster in females than in males, even when a lower level of alcohol has been consumed [121].

All in all, it seems evident that alcohol affects the amplitude and latency of various ERPs differently, particularly in young males and young females. P3 appears to be particularly sensitive to alcohol misuse, with males who abuse alcohol typically exhibiting lower amplitudes across the scalp. However, there may be differences in the frontal lobe, where females tend to show lower amplitudes, highlighting a significant sex difference that warrants further research attention. Investigating the heritability and genetic predictability of P3 could also be valuable, considering the notable sex differences observed and the influence of genetics and shared environment on each sex. While other ERPs like N415, P640, and ERN lacked sufficient methodological support to draw conclusions regarding sex differences, N340 and P540 suggest avenues for expanding research into memory processing, indicating differences in amplitude between males and females in response to various stimuli.

#### 4.2.3 AUD

Finally, the females with AUD were found to have significantly higher energy values than the males in the delta, theta, and beta frequencies in response to the alcohol-related stimuli compared with the non-alcohol-related stimuli [100]. EROs to alcohol-related stimuli may be biomarkers of comorbidities with AUD, particularly depression or anxiety, which is often observed in females with addiction problems [96, 122].

### 4.3 Clinical Applicability and Future Investigations

Overall, EEG techniques offer a cost-effective and portable solution beyond laboratory settings owing to their continuous technological advancements and improvements. They enable epidemiological studies, such as prospective cohort evaluations of AUD, as well as treatment-related progress and outcomes [123]. Moreover, by identifying any possible sex differences in the effects of alcohol consumption on EEG, clinicians could adjust treatment approaches to better suit the individual needs of young people [59]. With regard to this, electroencephalogram-neurofeedback (EEG-NF) is a tool that has experienced renewed interest in both clinical and research areas and has demonstrated positive and promising effects on inhibition and attentional skills [124]. Unfortunately, there is no literature about sex-related effects on EEG-NF parameters involved in AUD, but recent studies have examined possible brain electrical activity targets involved in cognitive processes altered in AUD. Anomalies in the N200/P300 complex component, characterized by either increased or decreased amplitudes and prolonged latencies, are commonly noted, indicating disrupted oscillatory activity. A wealth of experimental studies exploring the time-frequency features of ERPs suggests that dysfunctional fronto-central theta (3-7 Hz) and centro-parietal delta (< 3 Hz) frequencies underlie this alteration in P300 amplitude during cognitive processing of inhibitory stimuli in tasks such as Go/NoGo. Consequently, delta-theta NF protocols are frequently employed in research analyses due to their potential to address inhibitory control issues, presenting promising avenues for further investigation [125]. In contrast, most of the papers analysed used the alpha/theta protocol to reduce the 'hyperexcitation' of the nervous system. This protocol provides relaxation, decreases anxiety or stress, prevents alcohol relapse, maintains abstinence, and increases the feeling of well-being [126, 127]. Together with this, from a preventive standpoint, the findings of the review could assist in the development of programmes specifically targeted at youth, considering sex differences in the effects of alcohol on the brain. For instance, EEG data combined with other tools, such as self-report or cognitive assessment, could help during the initial phase to identify those young individuals who are at risk [128]. From a preventive perspective, there are several areas probably altered in risk individuals who are involved in alcohol misuse or pre-AUD. Among them, we can highlight incentive salience, negative emotionality, and problems in executive functions [129]. The most consistent findings have emerged from the analysis of ERPs, particularly focusing on the P3 component, which could have significant clinical utility, primarily in addressing deficits in executive functions. Additionally, there are other components involved in affective and substance-related processing (P1, N1, or the late positive potential LPP), along with event-related oscillations like theta power, which might serve as potential vulnerability or clinical markers in AUD. Moreover, novel tools arising from psychophysiology research, leveraging functional connectivity or brain graph analysis, could contribute to a deeper understanding of altered circuits in alcohol misuse and AUD [130]. On the other hand, heightened reactivity to threat may be a shared vulnerability factor for anxiety and alcohol abuse and a novel prevention and intervention target for anxiety-alcohol abuse comorbidity [130]. Regarding this, it has been observed that at high anxiety symptoms, greater alcohol misuse was associated with a more enhanced ERN [131]. Finally, it is also worth noting that EEG is currently experiencing a series of innovations, including its transformation into a wireless technology, and most notably its usage alongside virtual reality, which is bringing interesting findings in the field of AUD [131]. These findings should open the door for future research focused on the perspective of sex differences on the effects of drugs, especially alcohol, on the central nervous system.

With regard to the limitations of our review, we must highlight the following points. On the one hand, despite finding several articles on the topic, the literature is not very extensive regarding the specific analysis of sex differences. On the other hand, while some of the studies reviewed were aimed at finding any potential sex-related effects or differences, this was not the case for all of them, as they happened to unintentionally find those results, meaning that even if we could recover them, it does not guarantee that their reported effects were correctly controlled or adjusted. Additionally, although most articles assessed were of good quality, it should be noted that some did not have a high standard of methodological quality, potentially affecting the reliability and validity of the review results. On the other hand, the studies were highly heterogeneous in terms of the study population, type of alcohol consumption, EEG methods, and the cognitive paradigms evaluated, which complicates the comparison and synthesis of the results. Finally, we focused on young individuals, and many of the studies were conducted with university students, limiting the generalizability of the results to other groups.

## CONCLUSION

Our systematic review revealed that several of the reviewed ERPs (P3, N340, or P450, among others), together with resting state measures, could act as indexes of alcohol-related alteration of brain function. Differences based on sex were found, showing that alcohol affects brain function differently in both sexes. Similar findings were observed with regard to ERO and the resting-state paradigms, in which beta, theta, and gamma bands displayed significant differences with respect to alcohol and sex variables. All in all, it seems that with the inclusion of sex in clinical research, we could make advances in our understanding of sex-dependent alterations in brain function and structure with the goal of tailoring pharmacotherapeutic treatment strategies for AUD, particularly in young females.

## Figures and Tables

**Fig. (1):**
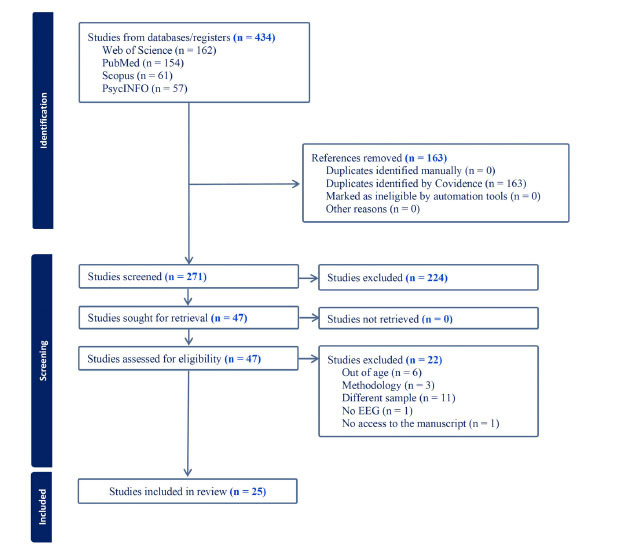
Flowchart of studies on EEG, youth, and alcohol.

**Table 1:** Quality assessment scores according to the NHLBI quality assessment tool for observational cohort and cross-sectional studies.

**Study**	**Q1**	**Q2**	**Q3**	**Q4**	**Q5**	**Q6**	**Q7**	**Q8**	**Q9**	**Q10**	**Q11**	**Q12**	**Q13**	**Q14**	**Quality**
Affan *et al*., 2018	Y	N	NR	Y	Y/N	N	N	N	Y	N	Y	NR	NA	N	Poor
Barrett *et al*., 2004	Y	Y	NR	Y	N	N	N	N	Y	N	Y	N	NA	N	Fair
Blanco-Ramos *et al*., 2019	Y	Y	NR	Y	Y/N	N	N	N	Y	N	Y	NR	NA	Y	Good
Criado *et al*., 2007	Y	Y	NR	Y	N	N	N	N	Y	N	Y	Y	NA	Y	Good
Ehlers *et al*., 2004	Y	Y	NO	Y	N	N	N	N	Y	N	Y	NR	NA	N	Poor
Ehlers and Phillips, 2007	Y	Y	NR	Y	N	N	N	N	Y	N	Y	Y	NA	N	Fair
Ehlers *et al*., 2011	Y	Y	NR	Y	N	N	N	N	Y	N	Y	Y	NA	Y	Good
Ehlers *et al*., 2019	Y	Y	NR	Y	N	N	N	N	Y	N	Y	Y	NA	Y	Good
Harper *et al*., 2018	Y	Y	NR	Y	N	N	N	N	Y	N	Y	NR	NA	N	Fair
Huang *et al*., 2018	Y	Y	NR	Y	N	N	N	N	Y	N	Y	NR	NA	Y	Poor
Kamarajan *et al*., 2015a	Y	Y	NR	Y	N	Y	N	N	Y	N	Y	NR	NA	N	Fair
Kamarajan *et al*., 2015b	Y	Y	NR	Y	N	Y	N	N	Y	N	Y	NR	NA	N	Fair
Kinreich *et al*., 2021	Y	Y	NR	Y	N	N	Y	N	Y	N	Y	NR	NA	N	Fair
Kiss *et al*., 2023	Y	Y	NR	Y	N	N	Y	N	Y	Y	Y	NR	NA	Y	Good
Magee *et al*., 2023	Y	Y	NR	Y	Y	N	N	N	Y	N	Y	NR	NA	Y	Good
Maurage *et al*., 2009	Y	Y	NR	Y	N	Y	Y	N	Y	Y	Y	NR	Y	Y	Good
Meyers *et al*., 2019	Y	Y	NR	Y	Y/N	N	Y	N	Y	Y	Y	NR	NA	Y	Good
Perlman *et al*., 2009	Y	Y	NR	Y	Y/N	N	N	N	Y	N	Y	NR	NA	N	Poor
Perlman *et al*., 2013	Y	Y	NR	Y	Y/N	Y	N	N	Y	Y	Y	Y	NA	N	Good
Petit *et al*., 2013	Y	Y	NR	Y	Y/N	N	N	N	Y	N	Y	NR	NA	Y	Good
Rangaswamy *et al*., 2004	Y	Y	NR	Y	N	N	N	N	Y	N	Y	NR	NA	N	Poor
Smith *et al*., 2015	Y	Y	NR	Y	Y/N	N	N	N	Y	N	Y	NR	NA	N	Fair
Smith *et al*., 2016	Y	Y	NR	Y	Y/N	N	N	N	Y	N	Y	NR	NA	N	Fair
Smith *et al*., 2017	Y	Y	NR	Y	Y/N	N	N	N	Y	N	Y	NR	NA	N	Fair
Yoon *et al*., 2006	Y	Y	NR	Y	N	Y	N	Y	Y	N	Y	NR	NA	Y	Good

**Table 2:** Summary of the 25 studies included in the systematic review, their methodology, and main findings.

**Study**	**Sample**	**Age ** **(Mean ± SD)**	**Alcohol Consumption Criteria**	**Inclusion & ** **Exclusion Criteria**	**Task**	**Sex-related Findings**	**Conclusions**
Affan *et al*., 2018 Cross sectional in United States of America [80]	61 (30 ♀) of which AUD = 30 (15 ♀)	23.4 ± 3.4	Binge Drinking (BD) = >6/2 h (males) and >5/2 h (females). Self-Administered Short Michigan Alcoholism Screening Test, (SMAST).	Inclusion: Control and BD. Exclusion: Tobacco or illicit drug use, history of neuropsychiatric conditions or brain injury or medication.	Resting-state with eyes-open and eyes-closed conditions.	No relevant effects or interactions were found.	BD during youth was associated with dysregulation of the spontaneous electroencephalography (EEG) signal, which was reflected in the slowing down of alpha peak, along with an increased power in theta and beta bands.
Barrett *et al*., 2004 Cross sectional in United Kingdom [100]	12 (12 ♀)	20.7 ± 0.1	Regular but not excessive alcohol drinkers (Mean: 20.2 units per week)	Inclusion: Experienced drivers, good sleepers, regular drinkers. Exclusion: Current medication, smokers, unexperienced drivers, no good sleep habits and not low or moderate caffeine consumption.	Driving simulation	No significant difference between conditions for 4-11 Hz power, unlike males. Females had more beta (14-26 Hz) power during both alcohol conditions compared with males.	The legal Blood Alcohol Concentration levels worsened sleepiness-impaired driving in females. Fortunately, they were generally aware of their impaired driving and assess its risk. This attitude may be the reason behind the lower incidence of sleep or alcohol-related crashes in females compared with males.
Blanco-Ramos *et al*., 2019 Cross sectional in Spain [87]	151 (81 ♀) of which AUD = 71 (43 ♀)	18-19	5/7(females/males) standard drinking units (SDU; 10 g of alcohol, according to Spanish definition of the SDU) on one occasion, raising blood alcohol concentration above 0.08 g/dl. AUDIT-C	Exclusion: Chronic pathologies, history of neurological disorder/brain injury with loss of consciousness >20 min. Personal/Family history of diagnosed psychopathologies. SCL-90-R score > 90^th^ percentile on GSI or ≥ 2 symptoms. Family history of first-degree AUD/SUD. Regular consumption of psychoactive drugs. Use of illegal drugs (except cannabis) in the last 6 months. Non-corrected sensory/motor deficits.	Go/NoGo task with alcohol and non-alcohol related stimuli.	ERP components: P1 and N2 had no sex differences, P3 differences between stimulus were only significant in females. For the group/sex/type of stimulus interaction, in Binge Drinkers (BDs) the difference was only significant in females.	BDs may need increased activation to monitor conflict to compensate for overactivation of the affective-automatic system caused by alcohol-related bias.
Criado *et al*., 2007 Cross sectional in United States of America [97]	222 (130 ♀) of which AUD = 53 (26 ♀)	18-30 23.3 ± 0.3	Semi-Structured Assessment for the Genetics of Alcoholism (SSAGA) according to DSM-IIIR criteria.	Inclusion: Hispanic heritage, lived within 25 miles, ages 18-30, able to read and write in English. Exclusion: Pregnancy or nursing, major medical or neurological disorder, head injuries that might bias the ERP testing. No alcohol or any other substance use for 24h prior to testing. No physical or behavioural signs of alcohol withdrawal.	Facial discrimination task.	Males had significantly diminished P450 responses, when compared to females which were further reduced in males with antisocial personality disorder/conduct disorder. P450 amplitudes were also significantly increased in males with high extraversion scores and in females with high neuroticism scores.	These data suggested that interpretations of P450 responses need to consider the relationship between sex, affective valence of the eliciting stimuli, as well as psychiatric status.
Ehlers *et al*., 2004 [82]	377 (242 ♀)	Hispanic Americans: 22.3 ± 0.3 Non-Hispanic Americans: 21.9 ± 0.2	DSM-IV criteria for AUD. Family History Assessment Module (FHAM). Semi-Structured Assessment for the Genetics of Alcoholism (SSAGA)	Inclusion: Must have consumed alcohol. History of abuse of drugs. Exclusion: Must have never met DSM-IV criteria for dependence on alcohol or an illicit substance, good health, no chronic medication. Current diagnosis of abuse of drugs.	Eyes-closed resting state EEG.	Compared with males, females drank less frequently and drank less drinks per occasion. In comparison with males, females had higher overall power in the low frequency alpha (7.5-9 Hz) and beta (12-20 Hz, 20-50 Hz) frequency ranges.	EEG measures and indices such as ethnicity, family history or alpha’s voltage were not as good at predicting AUD, as previously thought, but sex differences in EEG were detected.
Ehlers and Phillips, 2007 Cross sectional in United States of America [83]	237 (139 ♀) of which AUD = 61 (31 ♀)	AUD: 24 ± 4 Control: 23.1 ± 3.8	DSM-IIIR criteria. Family history of AUD was assessed using the Family History Assessment Module.	Pregnancy or nursing, major medical or neurological disorder, head injury that might bias the EEG testing.	Resting state EEG.	Significant relationship between sex and the three alpha variants. No male participants with AUD with high alpha variants. AUD, but not a family history of AUD, was associated with lower spectral power in the alpha frequency range.	Male sex and AUD were associated with an absence of high-voltage alpha variants and lower alpha power in the EEG. These data suggested that EEG low voltage, a highly heritable trait, may represent an important endophenotype in male Mexican Americans. that may aid in linking brain function with genetic factors underlying AUD in this ethnic group.
Ehlers *et al*., 2011 [98]	340 (205 ♀) of which AUD = 89 (47 ♀)	23.5 ± 3.7	(same as above)	(same as above)	ERP Acoustic startle stimuli.	Analysis of the late 400ms wave (N4S) show that females had significantly higher amplitude N4S responses to the startle and pre-pulse startle stimuli. Females also showed an increase in the eye blink response to startle and pre-pulse startle stimuli.	Sex and a lifetime diagnosis of AUD may contribute to the frontal late wave electrophysiological response to startle stimuli.
Ehlers *et al*., 2019 [101]	580 (359 ♀) of which AUD = 271 (136 ♀)	23.62 ± 3.8	Semi-Structured Assessment for the Genetics of Alcoholism. DSM-5 AUD criteria	(same as above)	Alcohol *vs*. non-alcohol visual stimuli reaction time.	Females displayed significantly higher energy values in response to alcohol-related stimuli than males in several regions: FZ for delta, theta and beta frequencies, CZ for theta and beta frequencies and in PZ in beta frequency. Phase synchrony showed that males had higher PLI synchrony than females when reacting to alcohol-related stimuli in the beta frequencies in CZ, and higher PDLI synchrony (FZ-PZ) in the delta and beta -frequencies. ERO energy and synchrony between the alcohol-related drinks and non-alcohol related drinks showed that males had larger differences in delta but smaller differences in PDLI synchrony in delta as well as smaller differences in PLI synchrony in alpha.	EROs may be biomarkers of comorbid risk factors, symptoms and disorders associated with AUD, adding the possibility to differentiate among those with “dark-side symptoms”.
Harper *et al*., 2018 Cross sectional in United States of America [102]	300 (185 ♀) of which AUD = 36 (14 ♀)	24.8 ± 0.7	Modified Substance Abuse Module to meet DSM-5 AUD criteria.	Inclusion: Monozygotic (MZ) and Dizygotic (DZ) twins. Exclusion: Not specified.	Go/No-Go task.	For females, greater alcohol misuse is associated with reduced NoGo MFC theta. Greater alcohol use was associated with reduced connectivity during NoGo trials, and that this effect was stronger for females than males. Females exhibited greater connectivity than males during go trials.	Diminished theta-band in the medial prefrontal cortex power and medial prefrontal cortex – dorsal prefrontal cortex connectivity may be neurophysiological mechanisms underlying alcohol-related disinhibition. Normative levels of alcohol use during emerging adulthood had potential sex-specific causal effects on response inhibition EEG dynamics.
Huang *et al*., 2018 Cross sectional in United States of America [103]	64 (32 ♀) of which AUD = 32 (16 ♀)	18-30 23.3 ± 3.3	BD = >6/2 h (males) and > 5/2 h (females). BD group = 5 episodes / 6 months	Inclusion: Control and BD. Exclusion: Drug use or cigarette smoking for at least 1 month prior to the study, history of seizures, brain injury, neurological or neuropsychiatric disorders, vision or hearing problems or learning difficulties, and were taking any medications at the time of the study.	Emotional rating task.	IAPS ratings were equivalent across groups. BD: Affective modulation of theta power ERO was attenuated during early appraisal and integrative processes, especially in high intensity BD.	BD may be associated with altered EEG indices of affective functions such as lower theta responsivity to emotions. This was consistent with compromised affective functions in AUD.
Kamarajan *et al*., 2015a Cross sectional in United States of America [88]	1864 (961 ♀) of which AUD = 1052 (800 ♀)	12-25	DSM-IV AUD. Semi Structured Assessment for the Genetics of Alcoholism. The Family History Assessment Module. Barratt Impulsiveness Scale.	Exclusion: Recent substance or alcohol use, hepatic encephalopathy/ cirrhosis, history of head injury, seizures or neurosurgery, uncorrected sensory deficits, history/ symptoms of psychosis, self-reported positive test for human immunodeficiency virus, acute/ chronic medical illnesses that affects brain function.	Monetary gambling task.	Males had significantly higher P3 amplitude than females only at the parietal region during the gain condition, but only in the younger age group. Overall P3 amplitudes were higher for males in central, parietal, and occipital regions, but the frontal region had the opposite pattern (females > males). Males displayed higher P3 amplitudes than females in all but the older high-risk (HR) subgroup.	Older male and younger female’s HR offspring, had reward processing deficits detected through lower P3 amplitude and weaker Current Source Density activity, along with higher prevalence of externalizing disorders and higher impulsivity scores.
Kamarajan *et al*., 2015b Cross sectional in United States of America [104]	1821 (947 ♀) of which AUD = 1535 (789 ♀)	(Same as above)	(Same as above)	(Same as above)	(Same as above)	Younger males showed highly significant increases in theta, compared to younger females, but older females had higher theta power than older males. In the loss condition, males showed higher theta than females. Males had higher theta power than females only during the loss condition at frontal and central regions. Males had higher theta power during loss compared to gain in frontal regions and an opposite pattern in, parietal and occipital region. Females displayed significantly more theta power for gain than for loss in central, parietal, and occipital regions.	Theta power was usually found to be lower not only in people with AUD, but also in HR subjects when processing reward. It was proposed then, that reduced reward-related theta power, in addition to impulsivity and externalizing features, may be related to a predisposition to develop AUD and other related disorders.
Kinreich *et al*., 2021 Cross sectional in United States of America [84]	656 (280 ♀) of which AUD = 328 (140 ♀)	12-30	AUD group was defined as those diagnosed as unaffected during the first visit and diagnosed with lifetime DSM-5 AUD during a follow-up visit.	Inclusion: participants who were unaffected at their first visit. meet both the diagnostic criteria for AUD (by DSM-IIIR criteria and the criteria for definite alcoholism specified by Feighner *et al*. Exclusion: Not specified.	Eyes-closed resting state.	Higher accuracy rates for females compared with males for both ancestries. The set of features better predicted AUD females than males. AUD exhibited lower occipital gamma amplitude compared to control. AUD genders shared lower gamma parietal interhemispheric coherence (male) and amplitude (female). Higher theta in AUD males interhemispheric connectivity in the occipital, frontal and temporal lobes while females had higher slow wave intrahemispheric connectivity (delta, alpha) in frontal-parietal and temporal-parietal lobes.	Predicting vulnerability and identifying related predisposition biomarkers hold enormous possibilities including preventions tactics, treatments or simply avoidance. Paired with ancestry, sex, and age in the calculation of model prediction of the development of AUD.
Kiss *et al*., 2023 Longitudinal in United States of America [81]	94 (40 ♀)	12-22 15.1 ± 2.3	Customary Drinking and Drug use Record. National Consortium on Alcohol and Neurodevelopment in Adolescence modified Cahalan *et al*. classification.	Inclusion: No/low drinkers at baseline. Exclusion: Medical conditions or current/past severe psychiatric disorders, medications known to affect sleep or the central nervous system, sleep, apnea, periodic limb movement disorder, narcolepsy.	Polysomnography.	Alcohol misuse was associated with altered sleep continuity, architecture, and EEG measures, some of those effects were dependent on age and sex. Young females started rapid delta power decline at 12.53 years, which was 1.2 years earlier than young males.	Alcohol misuse during youth was associated with altered sleep continuity, architecture, and EEG measures, some of those effects were dependent on age and sex. Alcohol could be behind the altered brain maturation processes involved in sleep-wake regulation.
Magee *et al*., 2023 Cross sectional in United States of America [89]	104 (66 ♀) of which AUD= 58 (37 ♀)	No Depression/No BD: 18.7 (.88) No Depression/BD: 19.9 (1.3) Depression/ No BD: 19.2 (1.6) Depression/ BD: 19.4 (1.2)	>5/session for males 6 > 4/session for females in the past year or received a score of ≥ 8 on the AUDIT.	Inclusion: Center for Epidemiological Studies Depression Scale (CES-D) score ≥16 or a sub-threshold diagnosis of depression on the Diagnostic Inventory for Depression (DID) or a diagnosis of depression on the DID. Exclusion: Participants with CES-D scores within 4 points above or below the cut-off were not invited to participate in the lab visit. Significant alcohol use and/or depressive symptoms or low levels of alcohol use and/or depressive symptoms were invited to take part in a laboratory visit.	Emotional Go/NoGo task.	Significant BD interactions for N2 and P3 components. Significant BD interaction for response bias.	Differences in early inhibitory control were observed across emotions based on trial type among depressed non-BDs, and these differences were attenuated in the presence of BD. Effects of depression on later inhibitory control were specific to non-BDs.
Maurage *et al*., 2009 Cross sectional in Belgium [90]	36 (22 ♀)	18.2 ± 0.4	75-item questionnaire, adapted from a binge-drinking habits questionnaire evaluating previous and future alcohol-drug consumption, family history of alcohol consumption, social integration and medical problems.	Exclusion: No positive family history of AUD, very low past alcohol consumption, total absence of past BD habits; total absence of past or current drug consumption (including tobacco and any medication), major medical problems, central nervous system disease (including epilepsy), auditory impairment, moderate or high depression-anxiety scores, history of psychiatric disorder.	Emotional auditory task.	No sex influence on the quantity of alcohol consumed at session or on the latency delays for P1 and P3b.	Short-term BD can produce marked cerebral dysfunction undetectable by behavioural measures alone. The observed latency abnormalities, similar to those observed in long-term AUD, constituted an electrophysiological marker of slowed cerebral activity associated with BD.
Meyers *et al*., 2019 Longitudinal cohort in United States of America [85]	2625 (1339 ♀) at the beginning, then 1931, later 1324, later 842, later 428, finally 8. (For this study 1426 (727 ♀)	12-22 17.7 ± 7.4	Semi-Structured Assessment for the Genetics of Alcoholism. DSM-5.	Inclusion: HR families. Exclusion: Not specified.	Eyes-closed resting state.	AUD Polygenic Risk Scores (PRS) was associated with increased fronto-central, tempo-parietal, centro-parietal, and parietal-occipital interhemispheric theta and alpha connectivity in males but not in females. Phenotypic differences between males and females in EEG coherence weren’t found despite the striking differences observed in the influence of AUD PRS on EEG coherence.	Measures of neural connectivity, neurocognitive performance and substance use behaviour can be used to understand of how genetic risk variants from large GWAS of AUD may influence brain function.
Perlman *et al*., 2009 Cross sectional in United States of America [94]	(Same as below)	10-12	DSM-IV AUD. Expanded Substance Abuse Module of the Composite International Diagnostic Interview (SAMCIDI-E)	(Same as below)	(Same as below)	Not accounted for	P3AR was an indicator of risk for alcoholism, independent of any negative effect of alcohol use on adolescent brain development.
Perlman *et al*., 2013 Longitudinal in United States of America [95]	1212 (619 ♀)	10-12 at the beginning, then 14, then 17. Final range 16-20.	Meeting criteria for at least one symptom of DSM-IIIR or DSM-IV abuse or dependence of alcohol. Modified version of the Diagnostic Interview for Children. SAMCIDI-E.	Inclusion: Same sex twins in a one-day drive trip from the research center. Exclusion: physically or mentally impaired.	Modified rotated heads task.	Sex was not statistically significant in any main analysis or post hoc test.	P3AR was a good indicator of a nonspecific predisposition for adolescent-onset substance misuse.
Petit *et al*., 2013 Cross sectional in Belgium [91]	56 (31 ♀) of which AUD = 29 (14 ♀)	18-27 21 ± 2.5	≥ 6 drinks/2 hours (10 g alcohol) at most 3-4/week.	Exclusion: major medical problems, conditions of the central nervous system, epilepsy, brain injury, visual impairment, past or current drug consumption (other than alcohol, cannabis, and tobacco), and alcohol abstinence.	Visual oddball.	BDs had increased P3 reactivity to alcohol related cues, this effect was bigger among males.	Alcohol cue reactivity could be a possible mechanism through which a HR population, BDs, and males in particular, were prone to develop alcohol misuse.
Rangaswamy *et al*., 2004 Cross sectional in United States of America [86]	375 (192 ♀) of which HR = 171 (77 ♀)	16-25 Mean HR males/ females: 19.6/19.9 Mean low risk (LR) males/ females: 19.6/20.2	Semi Structured Assessment of Genetics of Alcoholism (SSAGA)	Exclusion: Positive breath analyzer. Hepatic encephalopathy cirrhosis, acute chronic illness, history of head injury, seizures, or neurosurgical procedures. Uncorrected sensory deficits. Human Immunodeficiency Virus +. Medication that affects brain function. Psychoactive substances in the past 5 days. offspring of females with AUD.	Resting state EEG.	Increased beta power for offspring of males with AUD. HR males had higher beta 1 (12-16Hz) and HR females had increased power in beta 2 (16-20 Hz) and beta 3 (20-28 Hz) as compared with LR. HR Female who had two or more AUD first-degree relatives show significantly increased beta 2 and beta 3 when compared with HR females with only 1 affected father.	Risk characteristics expressed themselves differently in males and females and may be an index of vulnerability to AUD. These results indicated that increased EEG beta power can be considered as a likely marker of risk for developing AUD and may be used as a predictive endophenotype.
Smith *et al*., 2015 Cross sectional in Australia [92]	66 (32 ♀) of which AUD = 31 (15 ♀)	18-25	≥ 4 Australian standard drinks = 40 g alcohol. AUDIT. DUDIT-E.	Exclusion: Psychotropic medication, epileptic seizure, head injury or period of unconsciousness, uncorrected vision problems. >1/month use of any drug (apart from alcohol or tobacco) as assessed by DUDIT-E	Eriksen flanker task. Counterbalanced conflict monitoring task and error awareness task.	Conflict adaptation for N2 (indexing monitoring) was larger for female heavy drinkers than controls, and the opposite was observed for males. There were no interactions involving group or sex for the P3 (indexing inhibition).	A compensatory response was required to increase performance monitoring to achieve the same behavioural outcome as controls. Sex was an important factor in the relationship between behavioural control and heavy alcohol misuse.
Smith *et al*., 2016 Cross sectional in Australia [93]	71 (30 ♀) of which AUD = 34 (13 ♀)	-	N/LDs: no regular (less than once a month) consumption of ≥ 4 SADs/ occasion BDs: ≥ 4 SADs/occasion at least once a month preceding 12 months.	Exclusion: Epileptic seizure, serious head injury or LoC; Uncorrected hearing/vision problems; and Regular (≥ 2/ month) use of other drugs	Visual stop-signal task.	BDs: bigger P3 amplitude for successful inhibition trials. Longer P3 latency for failed inhibitions (only in females); lower Error Related Negativity (ERN) amplitude than N/LDs.	EEG deficits during response inhibition and performance monitoring seemed to be common in both sexes. Females also seemed to be more vulnerable at behavioural level.
Smith *et al*., 2017 Cross sectional in Australia [99]	Study 1: 33 (0 ♀) of which AUD = 12 Study 2: 104 (45 ♀) of which AUD = 29 (16 ♀)	Study 1: 16-18 Study 2: 18-21	Study 1: ≥ 4 Australian standard drinks, = 40 g alcohol. AUDIT. Study 2: (same as above) DUDIT-E	Study 1: Not regular users of any other drug apart from alcohol, cannabis, or tobacco. Normal or corrected vision. Psychoactive medications, seizure, or serious head injury. Study 2: no medication other than contraception or antibiotics. Psychiatric illness.	Study 1: modified Rey Auditory Verbal Learning Test (RAVLT). Study 2: RAVLT.	Study 1: No sex related differences were found. Study 2: male controls and heavy drinkers (HDs) had similar learning over trials, while female HDs had greater learning over trials than female controls. For females, proactive interference was greater for controls than HDs but the opposite was true for males. For the N340 males showed larger amplitudes to old words while females showed larger amplitudes to new words.	It was possible to measure meaningful and reliable ERP components in the RAVLT, and its sensitivity in detecting alcohol- and cannabis-related deficits not apparent in performance measures.
Yoon *et al*., 2006 Cross sectional in United States of America [96]	1100 (599 ♀) of which AUD= 499 (211 ♀)	17.5 [16.6-18.5]	Frequent use: ≥1 SDU/week in the past year. BD: ≥ 7 SDU at one time, at least once a week for a minimum of 2 months. Substance Abuse Module interview.	Inclusion: MZ or DZ population. Living with at least one biological parent and within a day’s drive the research centre. Exclusion: mental or physical disability.	Rotated-heads oddball paradigm	Heritability of P3 was approximately twice as great among males as among females, the influence of shared environment was significant only among females. For males 13 of the 15 comparisons were significant, which suggests an association between phenotype group and P3 amplitude. Mean reductions in P3 amplitude appeared relatively uniform across phenotype groups. For females, P3 amplitude was also reduced in all groups but one.	P3-AR phenotypes may provide tools for finding vulnerability genes in adolescents who have yet to pass through the age of risk for AUD.
**Total∑**	12571 (6615♀)	-	-	-	-	-	-

**Abbreviations:** AUD= Alcohol Use Disorder; AUDIT= Alcohol Use Disorders Identification Test; BD= Binge Drinking; BDs: Binge Drinkers; CES-D: Center for Epidemiological Studies Depression Scale; DID: Diagnostic Inventory for Depression; DZ=Dizygotic; EEG= Electroencephalography; ERN= Error Related Negativity; HDs: Heavy Drinkers; HR= High Risk; LR= Low Risk; MZ= Monozygotic; PRS= Polygenic Risk Scores; RAVLT= Rey Auditory Verbal Learning Test; SAMCIDI-E: Expanded Substance Abuse Module of the Composite International Diagnostic Interview; SDU= Standard Drinking Units.

## LIST OF ABBREVIATIONS

AUD: Alcohol Use Disorder
AUDIT: Alcohol Use Disorder Identification Test
BD: Binge Drinking
BDs: Binge Drinkers
BAC: Blood Alcohol Concentration
CNS: Central Nervous System
EEG: Electroencephalography
EEG-NF: Electroencephalogram-neurofeedback
ERN: Error-related Negativity
ERO: Event-related Oscillations
ERP: Event-related Potential
FC: Functional Connectivity
fMRI: Functional Magnetic Resonance Imaging
GABA: Gamma-aminobutyric Acid
HC: Hemispheric Connectivity
HR: High-risk
LR: Low-risk
NHLBI: National Heart, Lung, and Blood Institute
PET: Positron Emission Tomography
RAVLT: Rey Auditory Verbal Learning Test
SUD: Substance Use Disorder
TBR: Theta/Beta Ratio
WHO: World Health Organization

## STANDARDS OF REPORTING

PRISMA guidelines and methodology were followed.

## SUPPLEMENTARY MATERIAL

PRISMA checklist is available as supplementary material on the publisher’s website along with the published article.
