# Corrigendum to: “Effects of Alcohol on EEG Activity: A Systematic Review Focused on Sex-Related Differences in Youth”

**DOI:** 10.2174/1570159X23999250505094215

**Published:** 2025-05-05

**Authors:** Adrián S. Elliott, Román D. Moreno-Fernández, Patricia Sampedro-Piquero

**Affiliations:** 1 Departamento de Psicología Biológica y de la Salud, Facultad de Psicología, Universidad Autónoma de Madrid, Madrid, Spain;; 2 Facultad de Educación y Psicología, Universidad Francisco de Vitoria, Madrid, Spain

In the original publication of this article titled, “Effects of Alcohol on EEG Activity: A Systematic Review Focused on Sex-Related Differences in Youth”, published in Current Neuropharmacology, 2025, 06, 705-727 [1] the citation of Figs. (**2** and **3**) was missing in the text section.

Details of the error and correction are provided here.

**Original:**

Additionally, a seventh article using polysomnography was included in this section, and although it differed from the rest of the EEG resting state studies, we will also mention its main results here [80].

On the other hand, females, especially the HR females during the gain condition, exhibited significantly increased theta power compared to their male counterparts.

**Corrected:**

Additionally, a seventh article using polysomnography was included in this section, and although it differed from the rest of the EEG resting state studies (Fig. **2**), we will also mention its main results here [80].

On the other hand, females, especially the HR females during the gain condition, exhibited significantly increased theta power compared to their male counterparts (Fig. **3**).

The publisher regret the error and apologize to readers.

The original article can be found online at: https://www.eurekaselect.com/article/144423
